# Prenatal Alcohol Exposure Is Associated With Adverse Cognitive Effects and Distinct Whole-Genome DNA Methylation Patterns in Primary School Children

**DOI:** 10.3389/fnbeh.2018.00125

**Published:** 2018-06-26

**Authors:** Stefan Frey, Anna Eichler, Valeska Stonawski, Jennifer Kriebel, Simone Wahl, Sabina Gallati, Tamme W. Goecke, Peter A. Fasching, Matthias W. Beckmann, Oliver Kratz, Gunther H. Moll, Hartmut Heinrich, Johannes Kornhuber, Yulia Golub

**Affiliations:** ^1^Department of Child and Adolescent Mental Health, University Hospital Erlangen, Friedrich-Alexander University Erlangen-Nürnberg, Erlangen, Germany; ^2^Research Unit of Molecular Epidemiology, German Research Center for Environmental Health – Institute of Epidemiology II, Helmholtz Zentrum München, Munich, Germany; ^3^Division of Human Genetics, Department of Paediatrics, Inselspital University of Bern, Bern, Switzerland; ^4^Department of Obstetrics and Gynecology, University Hospital Erlangen, Friedrich-Alexander University Erlangen-Nürnberg, Erlangen, Germany; ^5^Department of Obstetrics and Gynecology, RWTH Aachen University, Aachen, Germany; ^6^kbo-Heckscher-Klinikum, Munich, Germany; ^7^Department of Psychiatry and Psychotherapy, University Hospital Erlangen, Friedrich-Alexander University Erlangen-Nürnberg, Erlangen, Germany; ^8^Department of Child and Adolescent Psychiatry, Faculty of Medicine of the TU Dresden, Dresden, Germany

**Keywords:** prenatal alcohol exposure, whole-genome DNA methylation, dipeptidyl peptidase 10 (DPP10), SLC16A9, attention, ERP, IQ, FRANCES

## Abstract

Prenatal alcohol exposure (PAE) is known to elicit a broad range of systemic effects, including neurophysiological alterations that result in adverse behavioral and cognitive outcomes. However, molecular pathways underlying these long-term intrauterine effects remain to be investigated. Here, we tested a hypothesis that PAE may lead to epigenetic alterations to the DNA resulting in attentional and cognitive alterations of the children. We report the results of the study that included 156 primary school children of the Franconian Cognition and Emotion Studies (FRANCES) cohort which were tested for an objective marker of PAE, ethyl glucuronide (EtG) in meconium at birth. Thirty-two newborns were found to be exposed to alcohol with EtG values above 30 ng/g (EtG+). Previously we described PAE being associated with lower IQ and smaller amplitude of the event-related potential component P3 in go trials (Go-P3), which indicates a reduced capacity of attentional resources. Whole-genome methylation analysis of the buccal cell DNA revealed 193 differentially methylated genes in children with positive meconium EtG, that were clustered into groups involved in epigenetic modifications, neurodegeneration, neurodevelopment, axon guidance and neuronal excitability. Furthermore, we detected mediation effects of the methylation changes in *DPP10* and *SLC16A9* genes on the EtG related cognitive and attention-related deficits. Our results suggest that system-wide epigenetic changes are involved in long-term effects of PAE. In particular, we show an epigenetic mediation of PAE effects on cognition and attention-related processes.

## Introduction

Adverse prenatal conditions have a potential to induce permanent changes in the developmental trajectories of the fetal brain. PAE is one of the most common *in utero* insults leading to a broad spectrum of structural, neurophysiological, cognitive and behavioral abnormalities (Hoyme et al., 2016). The degree to which alcohol affects development depends on a variety of factors including timing and the level of alcohol exposure (Pollard, 2007). At the most severe end of the spectrum is a FAS, which is described as a result of chronic exposure to high doses of alcohol (Jones and Smith, 1973; Del Campo and Jones, 2017). Whereas a clinical picture of FAS including pre- and postnatal growth retardation, facial dysmorphology, central nervous system alterations and intellectual impairment is well described, less is known about the impact of PAE that does not lead to the development of classic FAS. Newborns of mothers who consumed alcohol during pregnancy and seem to have no somatic abnormalities may reveal alterations in brain development (Flak et al., 2014). Cognitive and behavioral impairments and in particular attention deficits (Burden et al., 2005; Burger et al., 2011) in combination with hyperactivity, impulsivity (Mattson and Riley, 2000) and reduced inhibitory control have been described in children prenatally exposed to alcohol (Kingdon et al., 2016).

Prenatal adversity, such as substance exposure elicits its’ effects through long term alterations in the expression patterns of neurodevelopmental genes resulting in changed trajectories of both structural and functional brain development. A number of key studies have demonstrated that gestational alcohol exposure results in persistent genome-wide alterations to the transcriptome (Downing et al., 2012; Kleiber et al., 2012, 2013).

Increasing evidence suggests that epigenetic mechanisms are potential mediators linking environmental conditions; gene transcription and the phenotypic outcome (see for review, Murgatroyd and Spengler, 2012; Murgatroyd et al., 2015; Lussier et al., 2017). Indeed, numerous lines of evidence now point to epigenetic alterations in the etiology of FAS, including evidence from cell culture, various animal models, as well as a few clinical investigations on FAS (Rojas-Mayorquin et al., 2016; Lussier et al., 2017).

Three recent studies have characterized DNA methylation patterns of buccal epithelial cells of children with FAS (Bakdash et al., 2010; Laufer et al., 2015; Portales-Casamar et al., 2016). Laufer et al. (2015) identified a characteristic DNA methylation signature including novel genes and genes previously associated with alcohol exposure of the protocadherin cluster in children with FAS. Portales-Casamar et al. (2016) described 658 significantly differentially methylated sites between FAS cases and controls with enrichment for genes involved in neurodevelopmental processes and diseases, such as anxiety, epilepsy, and autism spectrum disorders.

Most studies on the effects of PAE investigated children with distinct FAS and/or based their work on a mother’s self-report regarding alcohol consumption during pregnancy (e.g., Furtado and Roriz, 2016), which is prone to systematic underreporting, due to retrospective recall bias and social stigmas (e.g., Eichler et al., 2016).

As a part of our FRANCES (Hein et al., 2014; Schneider et al., 2014; Eichler et al., 2016), we have studied attention and cognitive development of the 6- to 9-year-old children who were positive for EtG in meconium at birth, a child-related biomarker of intrauterine alcohol exposure (Eichler et al., 2018). Children with a positive meconium EtG, were found to have a 6-point lower IQ and a positive correlation between EtG value and ADHD symptoms (predominantly inattention) (Eichler et al., 2018). Considering ERPs in the EEG, which capture different aspects of attention and response control, EtG positive children (≥10 ng/g) were found to have a smaller P3 amplitude in go trials (Go-P3) indicating a reduced capacity of attentional resources.

In order to understand the mechanisms of these alcohol-mediated alterations, we compare the whole-genome DNA-methylation patterns in EtG positive children and children with no meconium EtG. Furthermore, we study how these epigenetic changes are related to the cognitive and attention-related deficits of the EtG positive children. Contrary to other studies all children included in the EtG-positive group did not fulfill the criteria of the FAS diagnose but were parentally exposed to measurable amounts of alcohol.

We hypothesized that (1) EtG values above the threshold are associated with differential genome-wide DNA methylation; (2) the effects of alcohol on IQ and the Go-P3 ERP component indexing attentional resources are mediated via alterations in gene methylation.

Indeed, we showed an association between meconium EtG with changes in the CpGs of 193 genes that were clustered into groups involved in epigenetic modifications, neurodegeneration, neurodevelopment and axon guidance, as well as neuronal excitability. In particular, methylation changes in the *DPP10* gene were associated with the IQ score and the differential methylation of several CpGs in the *SLC16A9* gene were linked to the Go-P3 ERP component that describes attention performance.

## Materials and Methods

### Study Design

**FIGURE 1:**
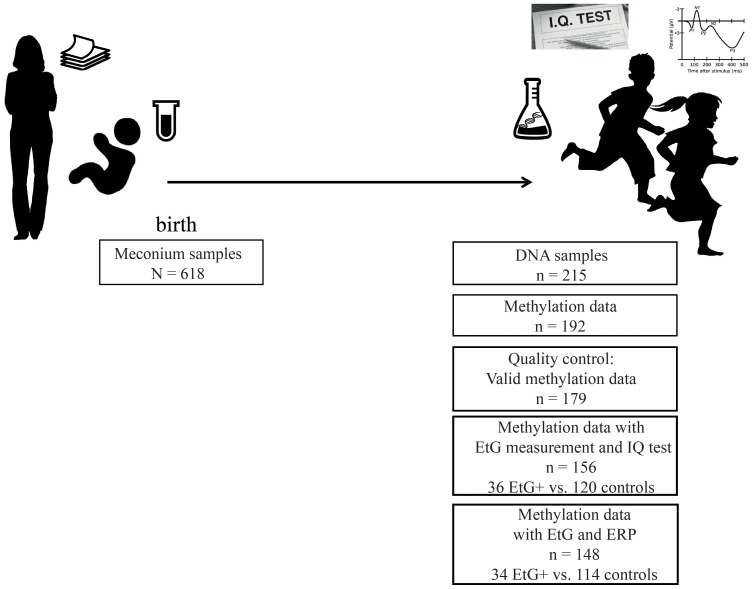
Schematic overview of the study design.

Our data was obtained from a subset of the longitudinal Franconian Maternal Health Evaluation Studies (FRAMES, Erlangen, Germany; Reulbach et al., 2009) and the follow-up study FRANCES (Eichler et al., 2016). During the first assessment in the years 2005 till 2007, 1,100 pregnant women older than 18 years were recruited. Perinatal maternal health data was collected during the third trimester (Reulbach et al., 2009; Goecke et al., 2014) at the Department of Obstetrics and Gynecology, University Hospital Erlangen, Germany. Mothers received information material including the statement that alcohol-related metabolites (e.g., EtG) will be analyzed in meconium samples of their newborns (**Figure 1**).

From 2012 to 2015, 618 families were contacted again for a follow up study. 198 families agreed to re-participate (35%) in the FRANCES study. The second assessment took place at the Department of Child and Adolescent Mental Health in Erlangen, Germany, where each child was tested for IQ and ERPs recorded during a cued Go/No-go task. Moreover, the information on maternal psychopathology and the socioeconomic status of the family was obtained during an appointment with trained psychologists who were blinded to the respective EtG results (**Figure 1**). The study design is described in detail in Eichler et al. (2017). Methylation sets of all participants with valid EtG measurements were divided into EtG + (EtG ≥ 30 ng/g) and control groups (**Table 1**).

**Table 1:** Sample characteristics and group differences of EtG+ children vs. controls.

	EtG + (EtG ≥ 30 ng/g)		Controls (EtG < 30 ng/g)				
					
					
	(*n*) (*Mean* ± *SD*)		(*n*) (*Mean* ± *SD*)		Test value	*p*	Effect size
Sex (m/f)	15/17		62/62		0.10^a^	0.753	-0.03^a^
Age (years)	32	7.44 ± 0.55	124	7.69 ± 0.53	2.01^b^	0.076	0.40^b^
Socioeconomic status	32	11.63 ± 1.89	124	11.12 ± 2.25	1.10^b^	0.270	0.22^b^
IQ	32	101.31 ± 10.05	124	105.64 ± 10.47	6.04^c^	0.015	0.04^c^
Abstract reasoning	32	9.50 ± 3.79	124	10.95 ± 2.70	2.03^b^	0.049	0.40^b^
Go-P3 (μV)	31	17.18 ± 5.13	117	20.62 ± 7.39	2.43^b^	0.004	0.49^b^


*Categorical variables were tested by ^*a*^Chi-Quadrat-Test, ^*b*^continuous variables by *t*-test, or ^*c*^ANCOVA. Test value: ^*a*^ χ^*2*^,^*b*^*t*, ^*c*^*F*; effect size: ^*a*^ φ, ^*b*^*d*, ^*c*^$\eta_{p}^{2}$.*

This study is consistent with the Declaration of Helsinki and was approved by the Local Ethics Committee of the University Hospital Erlangen (no. 4596). All parents gave written consent to this study and all children gave additionally their informed assent.

### Sample Characteristics

Two hundred and forty-five mothers with their children re-participated in the FRANCES study. DNA samples were obtained from 215 of their children. After exclusion of twins (*n* = 3), missing data (*n* = 7) and DNA samples with low quality (*n* = 13), 192 participants remained for the methylation analysis.

The 192 participating mothers did not differ in their marital status [χ^2^(1) = 0.42, *p* = 0.519], educational level [χ^2^(1) = 0.04, *p* = 0.856] or family income [χ^2^(2) = 2.58, *p* = 0.276] from the women who rejected the participation, nor did the group of children with EtG measurements [maternal marital status, χ^2^(1) = 0.42, *p* = 0.519; educational level χ^2^(1) = 0.04, *p* = 0.856; family income χ^2^(2) = 2.58, *p* = 0.276] at the time of birth. There were no significant group differences regarding child age, sex or socioeconomic status (**Table 1**).

Furthermore 13 methylation sets were excluded during pre-processing of the methylation data (for detailed information, see pre-processing of the methylation data), reducing the sample size to *n* = 179. After exclusion of missing EtG data (*n* = 23) the final data set comprised whole-genome methylation data of 156 children, among whom *n* = 32 children had meconium EtG ≥ 30 ng/g and *n* = 124 EtG < 30 ng/g. IQ data was available for all 156 methylation datasets (**Figure 1**). However, calculations with Go-P3 measurements were performed with a sample size of *n* = 148 due to missing Go-P3 data (*n* = 31 children with EtG ≥ 30 ng/g and *n* = 117 controls). DNA sampling, IQ-testing and recording of ERPs took place on the same day. Based on the recruiting strategy, risk factors were overrepresented in this cohort. All children included in the final methylation analysis (*n* = 179) were from single pregnancies and had a Caucasian ethnicity.

### EtG Measurement

Alcohol consumption during pregnancy was measured via the EtG- biomarker in 1 g meconium of the newborns. All samples were collected 2–24 h after birth. The subsequent EtG measurements were performed by Bakdash et al. (2010). EtG is a metabolic by-product of alcohol degradation commonly used to detect alcohol consumption (Palmer, 2009). About 75% of the meconium accumulates in the last 8 weeks of pregnancy in the fetal gut (Bakdash et al., 2010). Thus, positive EtG values represent an alcohol exposure during a time span of about 8 weeks (Bakdash et al., 2010).

The EtG value represents alcohol exposure but cannot be used for an estimation of the alcohol amount due to the individual metabolic rates. Furthermore, EtG measurements are sensitive to alcohol containing food and medication. To avoid false positives, we set the threshold for EtG+ group at 30 ng/g (Himes et al., 2015; Abernethy et al., 2017). Drinking self-report was not included as a variable in this analysis since earlier studies showed self-report results being inconsistent and less reliable than the EtG data (Lange et al., 2014; Eichler et al., 2016).

### Recording and Analyzing ERP

The Go-P3 component was measured in a motivational Go/No-go task which comprised four blocks of 36 trials each. Using Presentation (Neurobehavioral Systems, United States), visual stimuli were presented on a monitor. In go trials, a danger traffic sign (cue stimulus, S1) was followed by a green figure of pedestrian traffic lights (go stimulus, S2); for details, see Supplementary Figure S1. In the second and third task blocks, a monetary reward of 10 ct was given for fast responses in Go-trials to increase motivation (Supplementary Figure S1).

During the task, EEG activity was recorded from 25 sites (10–20 system plus additional midline electrodes and mastoid electrodes; recording reference: Fcz, ground electrode: CP2). Standard electrode caps with sintered Ag/AgCl electrodes (Easycap, Herrsching, Germany) were used for EEG activity measurement.

Electrooculogram electrodes were placed below and above the right eye and at the outer canthi. Filter bandwidth was set to 0.016–120 Hz; the sampling frequency was 500 Hz. Impedances were kept under 20 kΩ.

The following preprocessing steps were applied: downsampling to 250 Hz, filtering (bandpass: 0.5–20 Hz; 24 dB/Oct Butterworth filters and 50 Hz notch filter) and an ocular correction procedure (Gratton et al., 1983). Trials with performance errors (responses faster than 200 ms or slower than 1,500 ms) and amplitudes outside a range of ±150 μV were excluded from further analysis. Go-segments lasted from -150 to 1,150 ms (related to S2). Signals were re-referenced to linked mastoids. After signal averaging, the maximum amplitude of the Go-P3 component was determined at electrode Pz (where the Go-P3 showed its maximum) in a time window from 300 to 700 ms.

As Go-P3 effects of EtG-positive vs. EtG-negative children did not depend on the reward condition as described in Eichler et al. (2018), the Go-P3 averaged over blocks with and without incentives was used for the analysis in the present manuscript.

### Cognitive Development

Cognitive development was measured by standardized *IDS* (Grob et al., 2009). Total IQ score is based on the sum of seven subtest scores including visual perception, attention, phonological memory, spatio-visual memory, auditory memory, abstract reasoning and conceptual reasoning. The test is validated including gender- and age-specific norms, has an internal consistency of α = 0.92 and a retest-reliability (15 months) of *r*_tt_ = 0.83 (Grob et al., 2009). Testing was performed by experienced psychologists.

### Other Characteristics

Birth weight was recorded directly after delivery. Maternal smoking behavior was assessed by a self-report: here, ≥1 cigarette per day was set as a threshold for smoking.

Estimation of the socioeconomic status was based on maternal and paternal education level (4-level: <9, 9, 10, or 13 years) and net family income (6-level: <1,000 to >5,000) (sum-index, theoretical range: 3–14) as described in Geißler (1994).

Recording and analysis of the event related potentials as well as the methylation analysis procedure are explained in detail in the supplement.

### DNA Extraction and Methylation Analysis

DNA for methylation analysis was extracted from buccal cells, which were collected with OmniSwabs (Whatman^®^, kat. no. 28421853) from the children’s inner cheek. Subsequently, DNA was isolated with the QIAamp DNA Mini Kit (Qiagen, kat. no. 51306) according to the manufacturer’s protocol and stored at +4°C. 500 ng gDNA per sample was send to the Helmholtz-Zentrum München for genome-wide DNA methylation analysis. DNA methylation was analyzed using the Infinium Human Methylation 450K BeadChip array (Illumina).

### Pre-processing of Methylation Data

Pre-processing of methylation data were executed with R (version 3.2.2) using the packages *Minfi* (Aryee et al., 2014) and *wateRmelon* (Pidsley et al., 2013) according to Lehne et al. (2015) with minor modification. In particular, we analyzed all data sets for age and sex mismatches by shinyMethyl (Fortin et al., 2014) and DNAm age calculator (Horvath, 2013) prior to the data processing. Preprocessing was performed with the dataset of *N* = 179 samples. To account for technical variation in background fluorescence signals Illumina Background correction was applied to the raw intensity values. Subsequently, raw intensity values were normalized with quantile as implemented in *Minfi* and converted to beta values. These represent the proportion of DNA methylation at each single CpG. Probes were excluded if they fulfilled one of the following criteria: call rate < 97%, probes located on sex chromosomes or SNPs and a detection *p*-value > 0.001, which represents a low signal-detection rate from the background. Further exclusion criteria were mean beta <0.01 or >0.99 and a standard deviation less than 0.1 or above 4.0. To reduce technical bias we applied control probe adjustment to normalized beta values as recommended by Lehne et al. (2015). Control probe adjustment was done by a principal component analysis of the control probes with 22 factors explaining 95% of the variance (Supplementary Figure S3). Gender, age, maternal smoking behavior during pregnancy, and birth weight were added as cofactors to the regression model. The obtained residuals were used for a second PCA to explain further biological variance. The first two factors of the second PCA were added as predictors to a final regression model representing the values for further analysis. After exclusion of samples with missing EtG measurements (*n* = 23), 156 boys and girls were finally included in the analysis.

### Statistics

Differentially methylated CpGs were identified in an epigenome-wide analysis by independent *t*-tests (EtG+ group vs. control group) assuming unequal variances with the software R (version 3.2.2). A threshold of *p* < 10^-3^ was applied. To test a functional relevance of differentially methylated CpGs regarding total IQ score, the score of the abstract reasoning subtest, and Go-P3 we conducted three independent mediation models have been calculated using the SPSS macro PROCESS (version 2.16; Hayes, 2013). Our hypothesis was that alcohol exposure causes methylation changes, which in turn affect IQ, abstract reasoning, or Go-P3, which were tested by mediation analysis. Here EtG+ vs. control groups served as predictors, differentially methylated CpGs between these groups were considered mediators, and total IQ, the subtest abstract reasoning, or Go-P3 were considered outcome variables. Indirect effects were considered statistically significant at *p*-value < 0.05 as calculated by implemented Sobel test and bootstrapped 95% confidence intervals (CI) based on 10,000 samples without 0. In calculations containing total IQ or subset abstract reasoning as outcome variable, socioeconomic status was added as covariate to control for maternal and parental IQ. CpGs were included in the mediation analysis if they were identified by whole-genome analysis with a *p*-value less than 10^-3^ or located in a gene affected by the aforementioned criterion. A mediation model is a multilevel regression model between an independent variable, a mediator variable, and a dependent variable. In contrast to a direct relationship between the dependent and the independent variable the mediation model analyses an influence of the independent variable on the mediator variable, which in turn influences the dependent variable. The term “mediation effect” describes the two-level effect between independent variable and mediator as well as mediator and dependent variable. The “mediation effect” is represented by correlations between PAE and the methylation status and between the changes in methylation and IQ or Go-P3.

The second rule was solely applied if matching CpGs’ methylation status showed significant differences in a *t*-test between EtG+ and the control group.

If indirect models by Sobel test revealed a significant *p*-value, all CpGs of the respective genes were checked for methylation differences by a *t*-test. (Supplementary Table S2). Here, *p*-values were adjusted by FDR procedure (Benjamini and Hochberg, 1995). Additionally, thresholds for Bonferroni-correction of CpGs per gene were calculated.

Functional gene clustering was performed with online tool provided by the Database for Annotation, Visualization and Integrated Discovery (DAVID, v6.7; Huang da et al., 2009a,b).

The classification stringency used for clustering was set to medium (as recommended) and contained the following parameters: similarity overlap = 3, similarity threshold = 0.5, initial group membership = 3, and multiple linkage threshold = 0.5. DAVID calculated 68 clusters ranging between an enrichment score of 0.08 and 1.89.

## Results

### EtG+ Children Reveal Lower IQ Test Results and Alterations in the Attention-Related ERP

Using the FRANCES cohort, Eichler et al. (2018) showed IQ differences in children with an EtG concentration in meconium above the 50th percentile. Here, we report this effect to be also persistent in the group of the EtG positive children with a threshold set at ≥30 ng/g.

Controlling for socioeconomic status EtG+ children (*n* = 32) had a lower total IQ than controls (*n* = 124; *F* = 6.04, *p* = 0.015, $\eta_{p}^{2}$ = 0.04).

Looking at the subtests of the IDS, a significant effect was obtained for abstract reasoning (*t* = 2.03, *p* = 0.049, *d* = 0.40), which reflects the aptitude or ability to reason logically in non-verbal tests.

Moreover, the observed differences in the Go-P3 (Eichler et al., 2018) as reported recently were also stable using the threshold of EtG ≥ 30 ng/g (**Table 1**). The differences between the EtG+ group (*n* = 31) and controls (*n* = 117) were analyzed by *t*-test (*t* = 2.43, *p* = 0.004, *d* = 0.49). Here, one child of the EtG+ and 7 of the control group were excluded due to the insufficient quality of the ERP data.

### EtG+ Children Show Differential Genome-Wide DNA Methylation

After data processing, we obtained a list of CpGs and corresponding genes that showed significant differences in methylation between EtG+ and controls. The list was filtered down to stringent (*p* < 10^-3^, Supplementary Figures S4, S5), but not FDR corrected differentially methylated CpGs (193 genes in total, Supplementary Table S1). A *p*-value of 0.001 with the said sample size corresponds roughly to a medium effect size of 0.65 (Cohen’s *d*).

### Gene Ontology (GO) Results

To get insights in the affected biological processes we clustered our results into groups based on the functional annotations provided by the DAVID (v6.7; Huang da et al., 2009a,b).

In summary, the affected genes belonged to several groups primarily involved in regulating epigenetic modifications (SET Domain), neurodegeneration and development including cell growth and axon guidance as well as neuronal excitability. Moreover several genes involved in nucleotide binding, cell adhesion, transmembrane transport and exopeptidase function were affected by methylation changes (**Table 2**). 19% of these identified genes function in neuronal processes (Supplementary Figure S2). According to the analysis, the SET Domain group was enriched the most followed by nucleotide binding, cell adhesion and neuron differentiation.

**Table 2:** Functional groups of genes that show alterations in methylated CpG sites (*p* < 10^-3^) between EtG+ (≥30 ng/g) and control children.

Functional group	Hits	Enrichment	*p*-Value	Genes
SET domain	4	1.89	1.4E-2	*MLL4, PRDM2, PRDM12, PRDM16*
Nucleotide binding	31	1.86	3.4E-3	*AATK*, ***ABCC5***, *ACADSB, ACOX1, AGAP1, AK3, ATL3, DARS, DGKD, EIF2AK4, FARS2, GBP4, HCN3, HLCS, HSPA4, MASTL, MBD3, MCM6, MYH6, MYO9A, NLRP1, NWD1, PDXK, PIK3CD, PRKG1, RAB40B, SPAG1*, ***SRC***, *SSB, TOP3B, TTBK2*
Neuron differentiation	11	1.6	2.1E-2	*AGRN*, ***BAI1, CDH23, CTNNA2*,** *EMX1, MAPK8IP3*, ***NRP1*,** *PRKG1*, ***SEMA3B***, ***SLIT3, UNC5B***
Cell morphogenesis involved in neuron differentiation	8	1.6	7.8E-3	***BAI1, CDH23, CTNNA2, EMX1***, ***MAPK8IP3, NRP1, SEMA3B, SLIT3***
Axon guidance	5	1.6	2.9E-2	***MAPK8IP3, NRP1, SEMA3B, SLIT3, UNC5B***



**Medium validity**				
Cell adhesion	15	1.44	2E-2	*BAI1, CADM1*, ***CDH23***, *CLDN16, CLDN8, COL11A2, CTNNA2, CTNND2, HAPLN2, ITGB2, LSAMP, MSLN, MSLNL, NRP1*, ***SRC***
Exopeptidase function	4	0.82	4.1E-2	*CPA2, DPP6, DPP10, XPNPEP3*
Transmembrane transport	12	0.78	3.0E-2	***ABCC5***, ***HCN3***, *LOC494141*, ***KCNQ5***, ***KCNG2***, *NUP93, SFXN3, SLC12A7, SLC16A9, SLC25A12, SLC25A22, SLC9A9*
Voltage-gated potassium channel activity	4	0.78	9.1E-2	***HCN3, KCNG2, KCNQ5*,** *KCTD15*


*Genes were clustered into groups based on the functional annotations provided by DAVID (v6.7). Gene enrichment is calculated by a modified Fisher exact as implemented in the program. Names in bold are listed in at least two categories. Medium validity marks groups with an enrichment score below the threshold of 1.5.*

### The Methylation Changes in the Genes *DPP10* and *SLC16A9* Mediate the Differences in IQ and Go-P3 in Gene-Specific Analyses

Our hypothesis was that the direct effect of alcohol exposure on IQ and Go-P3 can be (partially) explained by an indirect effect containing methylation changes.

**FIGURE 2:**
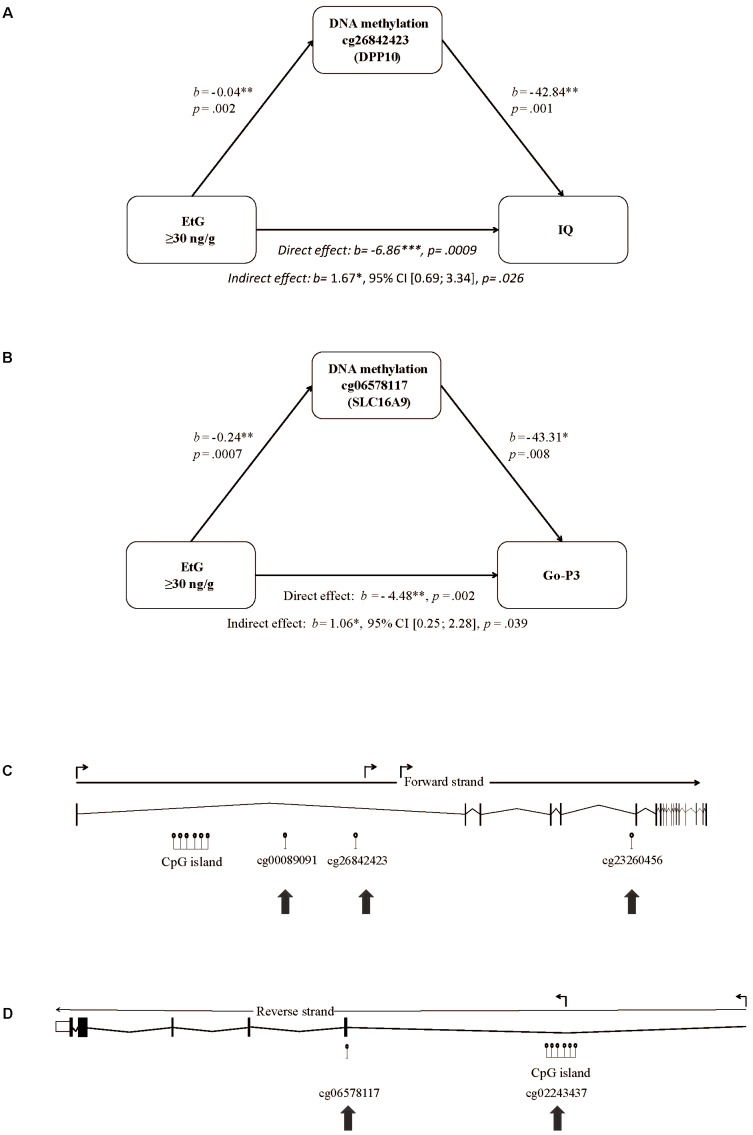
Mediation model of EtG as predictor for reduced **(A)** IQ, **(B)** Go-P3, and localization of the CpGs in the *DPP10* gene and *SLC16A9* affected by prenatal alcohol exposure. **(C)** The model consists of EtG as predictor, the methylation changes in cg26842423 (*DPP10*) between EtG+ (*n* = 32) and controls (*n* = 124) as mediator, and IQ as outcome. **(B)** The model consists of EtG+ as predictor, the methylation changes in cg06578117 (*SLC16A9*) between EtG+ (*n* = 31) and controls (*n* = 117) as mediator, and Go-P3 as outcome. The analysis was conducted with PROCESS (version 2.16; Hayes, 2013). Socioeconomic status was used as covariate. ^∗^*p* < 0.05, ^∗∗^*p* < 0.01, ^∗∗∗^*p* < 0.001. **(C)** Significant DNA methylation changes in the EtG+ group were observed in intron 1 and 5 of *DPP10* and **(D)** in the 5′UTR and exon 1 of the *SLC16A9* gene as indicated with arrows. Shown are the full-length genes with introns and exons according to UCSC (*http://genome.ucsc.edu, hg38;* Aken et al., 2016). The angled arrows above the transcription direction mark the positions for transcription variants.

We set EtG+ as predictor, the ethanol induced methylation changes of the 193 CpGs obtained by whole-genome analysis (Supplementary Table S1) as mediating variable, and IQ, its subtest abstract reasoning, or Go-P3 as outcome variable. The mediation analysis is a multilevel regression model revealing correlations between PAE, and the methylation status of the respective CpGs. Solely 2 of 193 CpGs were statistically significant in the indirect effect of the mediation analysis; CpG cg26842423 (*DPP10*) and CpG cg06578117 (*SLC16A9*). CpG cg26842423 (*DPP10*) could be identified as mediating variable for the association of EtG+, IQ (indirect effect*: b* = 1.67^∗^, 95% CI [0.69; 3.34], *p* = 0.026) and abstract reasoning (indirect effect: *b* = 0.46^∗^, 95% CI [0.17; 0.95], *p* = 0.030), respectively (**Figure 2A**). Another mediation analysis revealed the EtG-dependent (EtG ≥ 30 ng/g vs. controls) methylation of CpG cg06578117 (*SLC16A9*) as mediator for the effect of EtG on Go-P3 (indirect effect: *b* = 1.06^∗^, 95% CI [0.25; 2.28], *p* = 0.04*;* **Figure 2B**).

CpGs are known to be more likely methylated/demethylated in line with other CpGs. Thus, we investigated if other CpGs located in *DPP10* (*n* = 50) or *SLC16A9* (*n* = 22) were differentially methylated depending on the EtG+ threshold (Supplementary Table S2). Two additional CpGs in *DPP10* (4% of tested CpGs) and one more in *SLC16A9* (5% of tested CpGs) showed methylation differences between EtG+ vs. control group. None of these showed positive results in the mediation analysis with IQ, abstract reasoning or Go-P3 as outcome variable (Supplementary Table S2).

Moreover, CpGs’ positions determine its influence on the gene regulation and function. The positions of the aforementioned three CpGs of *DPP10* (cg26842423, cg00089091, and cg23260456) are shown in **Figure 2C**.

The second differentially methylated gene we report here is *SLC16A9*. CpG cg06578117 and cg02243437 are increased in methylation in the EtG+ group (Supplementary Table S2). The respective CpGs are both located in a region with enhancer function and thus may directly influence the expression of the *SLC16A9* gene. The positions of the CpGs of *SLC16A9* affected in the EtG+ group are shown in **Figure 2D**.

### *SLC16A9* and *DPP10* Are Indirectly Linked by Co-expression and Genetic Interaction

**FIGURE 3:**
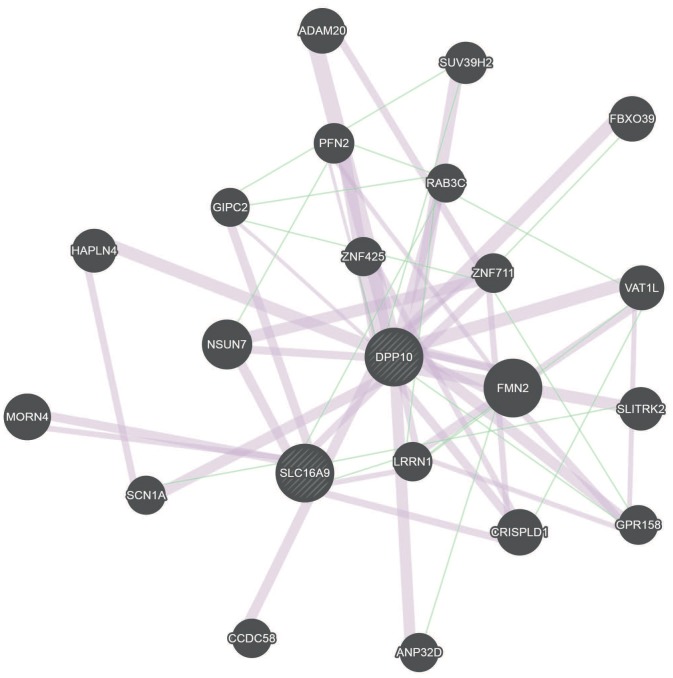
GeneMANIA network for *DPP10* and *SLC16A9* based on genetic interactions (green) and co-expression (red). *DPP10* and *SLC16A9* are indirectly connected over genetic interaction and co-expression.

Our study revealed that, alcohol exposure might influence children’s IQ and attentional capacity/Go-P3 by methylation changes in *DPP10* and *SLC16A9* genes. To identify possible links between these two genes, the geneMANIA network analysis was performed (**Figure 3** and Supplementary Figure S6). This analysis revealed a connection by co-expression and genetic interaction of *DPP10* and *SLC16A9* via expression of *LRRN1*, *CRISPLD1*, and *GIPC2* (Chen et al., 2002; Roth et al., 2006; Warde-Farley et al., 2010; Mallon et al., 2013).

## Discussion

Prenatal alcohol exposure is a major risk factor for adverse cognitive and behavioral phenotypes. Increasing evidence suggests that epigenetic mechanisms potentially mediate the effects of this *in utero* adversity on the neurodevelopmental processes (Lussier et al., 2017).

In the FRANCES cohort, we could previously demonstrate the negative impact of PAE on child’s cognitive development and ADHD-related behavior on the basis of the ethanol metabolite EtG in newborn’s meconium (Eichler et al., 2018). In this study, we present a comprehensive genome-wide analysis of DNA methylation in buccal cells of children of the same FRANCES cohort both positive and negative for meconium EtG. Furthermore, we examine the hypothesis whether the EtG associated effects on the cognitive and attentional processes are mediated by alterations in DNA methylation. In contrast to other studies, we focused on methylation changes in children without diagnosed FAS but measurable PAE.

In children with PAE we report 193 genes with differentially methylated CpGs (*p* < 10^-3^), which could be clustered into nine biological groups playing a crucial role in the epigenetic processes, neuronal differentiation, and neuronal excitability.

Several genes belong to the SET – domain and to the “nucleotide binding” cluster, both containing components of the epigenetic regulatory machinery for neural growth and differentiation (Cui et al., 1998). In particular, the methyl-CpG-binding domain protein 3 (*MBD3*) seems to be relevant for maintenance and establishment of 5-hydroxymethylcytosin (5hmC) by interaction with Tet proteins (Yildirim et al., 2011), last being previously linked to FAS (Cheng et al., 2015). In line with the results of Laufer et al. (2015), we observe methylation changes in genes coding for cell adhesion and genes of the protocadherins family. Interestingly, mutations in the *CDH23* gene of the cell adhesion domain result in deafness (Woo et al., 2014) and hearing loss is closely associated with FAS (Church, 1987).

Among 193 differentially methylated genes of the EtG positive group, 11 have an important function in neuronal differentiation and axon guidance. For instance, *CTNNA2* and *EMX1* function as transcription factors and are implicated in the cellular identity of cortical neurons. *EMX1* deletion in mice results in a smaller hippocampus and a lack of the corpus callosum (Qiu et al., 1996). In addition, Lim et al. (2015) showed that *EMX1* functions in “cingulate callosal axon guidance” by regulating the expression of *NRP1*, which in turn, was also identified as differentially methylated in the EtG+ group. Differentially methylated are also several genes coding for the proteins of the voltage-gated potassium channels. These are widely distributed in all brain areas and play an important role in neuronal excitability. Data was provided suggesting that potassium channel gene *KCNQ1* may contribute to the shared risk for diminished processing speed and white mater integrity (Bruce et al., 2017).

Several hits detected in our study were previously associated with alcohol-related disorders. *PRDM2* gene, for instance, involved in neuronal differentiation, has recently been associated with compulsive drinking and alcohol self-administration in rats (Barbier et al., 2016). *NLRP1* gene was described to play a role in alcohol-induced inflammation in neuronal cells (Youm et al., 2013; de Rivero Vaccari et al., 2014). *CTNNA2* has previously been identified as a risk gene for alcohol addiction (Song and Zhang, 2014). *ITGB2* in rat was hypothesized to contribute to high alcohol drinking behavior (McBride et al., 2013). *SRC* signaling was linked to alcohol-induced neuroinflammation and neurodegeneration (Floreani et al., 2010).

Prenatal alcohol exposure has been shown to perturb the epigenetic machinery in several animal (Tulisiak et al., 2017) and human studies (Laufer et al., 2015; Portales-Casamar et al., 2016). Laufer et al. (2015) characterized DNA methylation in a discovery cohort consisted of six FAS patients and five matched controls and validated their results in a replication cohort of six different FAS patients and six controls. They identified 269 significant (*p* < 0.005) differentially methylated CpG sites, in genes related to protocadherins, glutamatergic synapses, and hippo signaling (Laufer et al., 2015). An involvement of such functional clusters as neurodevelopment and axonal guidance, as well as cell adhesion, was observed in our study and in the study of Laufer et al. (2015).

Another recent work of Portales-Casamar et al. (2016) described 658 significantly differentially methylated sites between FAS cases and controls with enrichment for genes involved in neurodevelopmental processes. A further study validated 161 of these as possible predictors for FAS (Lussier et al., 2018).

Within the 193 differentially methylated genes of the EtG positive group two genes are associated with the IQ and alterations in the attention-related processes. In particular, our mediation analysis showed that the methylation of the CpG cg26842423 in the *DPP10* gene influences total IQ and abstract reasoning capacity. DPP10 is a single-pass type II membrane protein that binds specific voltage gated potassium channels. Thus, it plays an important role in a variety of cellular processes, such as neurotransmitter release, neuronal excitability and repolarization of action potentials. DPP10 has been linked to ADHD (Lasky-Su et al., 2008) and neurodegenerative processes in Parkinson’s and Alzheimer’s diseases (Chen et al., 2014). Several findings support DPP10 to be a target for multiple toxins, such as nicotine, alcohol, and phthalates (Blakey et al., 2009; Wu et al., 2010; Chhabra et al., 2014; Machtinger et al., 2018). More than 50 potential targets for DNA methylation have been identified within this gene so far. Among those Heinrich et al. (2017) reported an association between CpG cg19651219 (*DPP10*) with the Cue-P3, reflecting attentional orienting in ERPs, and ADHD behavior using the same cohort.

Consistently recurrent CNVs in the *DPP10* are enriched in autism-spectrum-disorders (Marshall et al., 2008; Girirajan et al., 2013). Interestingly, as the size of deletions increases, non-verbal IQ significantly decreases, but there is no impact on autism severity; and as the size of duplications increases, autism severity significantly increases but non-verbal IQ is not affected (Girirajan et al., 2013).

Other authors reported associations between PAE and ADHD symptoms (Mick et al., 2002; Knopik et al., 2006, 2009). It is important to mention that Eichler et al. (2018) found evidence of ADHD symptoms derived from PAE to be related to different pathways than ‘classical’ ADHD. This might explain the difference in affected CpGs in *DPP10* between ADHD and PAE. Interestingly, Knopik et al. (2016) also published an association between prenatal smoke exposure and ADHD symptoms. One explanation could be that *DPP10* is a key target for multiple prenatal risks connected to ADHD. This seems quite realistic since DPP10 is a peptidase with a broad range of functions that modulate the electrophysiological properties of voltage-gated potassium channels via Kv4 subunit [primary K(+) channel pore-forming subunit], and plays a crucial role in the neuronal excitability. The Kv4 subunit contributes to the somatic and dendritic A-type currents regulating of neuronal excitability and dendritic processing of incoming synaptic information. It was repeatedly shown to be involved synaptic plasticity and learning and memory (Alexander et al., 2009). In particular, deletion of Kv4.2 gene eliminates dendritic A-type K+ current and enhances induction of long-term potentiation in hippocampal CA1 pyramidal neurons (Chen and Johnston, 2006; Chen et al., 2006). Lugo et al. (2012) show Kv4.2 knockout mice having hippocampal-dependent learning and memory deficits.

We propose the model that multiple toxins affect *DPP10* which in turn has an effect on cognition and behavior.

There is no data on the functional changes on the expression regulation of the *DPP10* gene through the affected CpG (cg00089091 and cg26842423). All affected CpGs are located within introns. Several authors suggested that DNA methylation in the gene body could increase gene expression by blocking the initiation of intragenic promotors (Maunakea et al., 2010). Moreover, gene body methylation is also hypothesized to influence splicing and thus, defining splice variants (Laurent et al., 2010). Targeted cell culture studies are necessary in order understand the functional significance of this methylation change.

Previously we described, that children with a meconium EtG above the detection limit show reduced Go-P3 amplitudes, an ERP component that is related to the allocation of attentional resources and executive response control (Eichler et al., 2018). These results indicate a potential risk for specific attentional impairments (Spronk et al., 2008), although no other ADHD related neural or neuropsychological function was negatively influenced in EtG+ group. Several other studies found an association between maternal alcohol use during pregnancy and offspring ADHD symptoms (Infante et al., 2015; Eilertsen et al., 2017). Even after controlling for the impact of the postnatal environment, elementary school children, who experienced PAE, are highly vulnerable to develop mood disorders, anxiety disorders, ADHD, oppositional defiant disorder or conduct disorder (Burger et al., 2011).

Here, we report positive meconium EtG as a predictor of Go-P3 by cg06578117 (*SLC16A9*) methylation. *SLC16A9* is a monocarboxylate transporter and member of the soluble carrier family 16. This gene changes carnitine levels in blood by functioning as carnitine efflux transporter (Suhre et al., 2011). *SLC16A*9 was hypothesized to be a risk gene for neurodegeneration (Gonzalez-Aramburu et al., 2013). Sutin et al. (2014) showed a correlation between uric acid concentrations and impulsivity in humans and mice. Other studies found a positive correlation between uric acid concentrations and ADHD symptoms and thus uric acid may play a role in hyperactivity (Barrera et al., 1988; Johnson et al., 2011). Since SLC16A9 as carnitine efflux transporter directly influences the uric concentration, its activity could possibly influence ADHD symptoms.

The geneMANIA network analysis revealed an indirect connection between *DPP10* and *SLC16A9* by co-expression which is linked via *LRRN1*. *LRRN1* has previously been identified as neurite growth regulator in early development (Buchser et al., 2010).

Interestingly, aside from co-expression, *DPP10* and *SLC16A9* also genetically interact (Lin et al., 2010) via the Solute Carrier Family 9 member *SLC9A9*, which has also been identified as differentially methylated in our EtG+ group (Supplementary Table S2). *SLC9A9* functions an ion transport and is a risk gene for ADHD (de Silva et al., 2003). This is consistent with findings, that FAS is accompanied by an increased risk for ADHD (Burd et al., 2003; Rojas-Mayorquin et al., 2016). This connection may offer a model how PAE and finally FAS are linked to the increased risk of ADHD.

The main limitation of our study is a modest sample size for detecting small effects in a genome-wide analysis. Second limitation is a missing replication cohort though at least some of the results are in line with findings of recent epigenome-wide studies on FAS. Third, no reliable data on the correlations of the EtG levels and an amount of alcohol consumed by the mother exist so far; thus not allowing to predict alcohol induced adverse effects depending on the consume amount/type. Forth, EtG levels can depend on the metabolism of both fetus and the mother, another variable that might be associated with the methylation changes detected. Fifth, we made substantial efforts to eliminate Type I errors by setting the discovery threshold at *p* = 10^-3^. However, investigating over 450,000 sites in a sample of *n* = 156, these efforts might not be sufficient especially as this sample size did not allow to correct for FDR (Supplementary Figures S4, S5). A threshold of *p* = 10^-3^ corresponding to a medium effect size of 0.65 (Cohen’s *d*) was applied instead. Sixth, we examined methylation changes in buccal cells samples that might not or not exactly reflect changes in the brain tissue, we are concerned with. However, easily available samples are favorable when searching for potential biomarkers of prenatal adversity.

## Conclusion

This study reports 193 differentially methylated genes in buccal cells of children with positive meconium EtG, an objective marker of maternal alcohol consume. Additionally we show mediation effects of the methylation changes in *DPP10* and *SLC16A9* genes on the EtG-related cognitive and attention-related alterations in IQ and ERP. Our data shows that maternal alcohol consumption has a long-term impact on epigenetic programming of key neurodevelopmental genes resulting in adverse cognitive and behavioral outcome. Further studies are needed to validate our findings and to identify associated pathways.

## Availability of Data and Material

Methylation data have been deposited in the ArrayExpress database at EMBL-EBI^1^ under accession number E-MTAB-6730.

## Author Contributions

SF, VS, YG, HH, and AE analyzed the data and/or interpreted the results. JKr and SW were involved in the 450k methylation analysis. TG, JKo, PF, and MB initiated and designed the FRAMES project. TG and PF supervised the clinical data acquisition in FRAMES. OK, GM, HH, and AE were responsible for the study design of FRANCES. SG contributed to the design of the epigenetic part of FRANCES. SF and YG wrote the manuscript. All authors reviewed the manuscript.

## Conflict of Interest Statement

The authors declare that the research was conducted in the absence of any commercial or financial relationships that could be construed as a potential conflict of interest.

## Footnotes

1 www.ebi.ac.uk/arrayexpress

## Abbreviations

ADHD: attention deficit hyperactivity disorder
CNVs: copy number variations
DPP10: dipeptidyl peptidase 10
ERPs: event-related potentials
EtG: ethyl glucuronide
FAS: fetal alcohol syndrome
FDR: false discovery rate
FRANCES: Franconian Cognition and Emotion Studies
Go-P3: event-related potential component P3 in go trials
IDS: Intelligence and Development Scales
IQ: intelligence quotient
PAE: prenatal alcohol exposure
SLC16A9: solute carrier family 16 member 9
