# Supplementary material for: Prenatal Alcohol Exposure Is Associated With Adverse Cognitive Effects and Distinct Whole-Genome DNA Methylation Patterns in Primary School Children
Source: Front Behav Neurosci. 2018 Jun 26;12:125. doi: 10.3389/fnbeh.2018.00125 (PMC6028559; doi:10.3389/fnbeh.2018.00125)
Supplement: Supplementary file 1 [file Data_Sheet_1.PDF]

Supplementary Material

Prenatal alcohol exposure is associated with adverse cognitive effects and distinct whole-genome DNA methylation patterns in primary school children

Stefan Frey[1*], Anna Eichler[1], Valeska Stonawski[1], Jennifer Kriebel[5], Simone Wahl[5], Sabina Gallati[7], Tamme W. Goecke[2,4], Peter A. Fasching[2], Matthias W. Beckmann[2], Oliver Kratz[1], Gunther H. Moll[1], Hartmut Heinrich[1,6], Johannes Kornhuber[3*], Yulia Golub[1*]

([*]co-senior-authorship)

1 Supplementary Figures and Tables

1.1 Tables

Table S1: Differentially methylated CpGs with a p-value < 10^{-3}. All hits have been considered for functional clustering (Table 2). No multiple testing was applied due to 450k/genome-wide Bonferroni threshold of 10^{-7}.

	Gene name	CpG	p-Value
1	TTBK2	cg08194650	7.14E-06
2	TARM1	cg21966754	8.42E-06
3	EMX1	cg17090593	9.59E-06
4	C1orf14	cg22450662	1.13E-05
5	ZNF706	cg16269364	2.02E-05
6	INPP5E	cg13741927	2.29E-05
7	SNRNP27	cg20337934	2.40E-05
8	RAB40B	cg15250633	2.51E-05
9	PARK2	cg26872907	2.60E-05
10	RASA3	cg09634726	3.02E-05
11	C6orf106	cg10519906	3.24E-05
12	PIK3CD	cg04287259	3.41E-05
13	ATXN7L1	cg03199646	3.82E-05
14	KCNG2	cg17174613	3.97E-05
15	SMAD3	cg01624571	4.24E-05
16	TSSC4	cg23594951	4.33E-05
17	MRPS25	cg18017531	4.60E-05

18	MRC2	cg08863844	4.62E-05
19	GTF2E2	cg18356330	4.77E-05
20	DPP6	cg18239417	4.80E-05
21	AATK	cg02460935	5.10E-05
22	CHRNA5	cg16751781	6.45E-05
23	CASZ1	cg16926809	6.91E-05
24	CLDN16	cg12982543	7.10E-05
25	MT1F	cg02527372	8.31E-05
26	PRDM16	cg26014413	8.93E-05
27	LSAMP	cg26033586	1.06E-04
28	PID1	cg17804911	1.07E-04
29	SIPA1L3	cg00344482	1.12E-04
30	ZNF253	cg26933068	1.12E-04
31	MMEL1	cg20776977	1.17E-04
32	SBNO2	cg00467278	1.27E-04
33	ITGB2	cg14112356	1.38E-04
34	C17orf62	cg14231704	1.43E-04
35	ZMYND15	cg01840020	1.46E-04
36	EPB41L3	cg16304950	1.48E-04
37	LOC494141	cg26558664	1.48E-04
38	KPNA6	cg10332202	1.59E-04
39	SSU72	cg10332202	1.59E-04
40	POFUT2	cg03515060	1.63E-04
41	DYSF	cg08972867	1.63E-04
42	MMRN2	cg15347156	1.64E-04
43	MAD1L1	cg17815546	1.75E-04
44	AK3	cg14288486	1.76E-04
45	RTN4IP1	cg05080777	1.90E-04
46	GABRB2	cg01138867	1.94E-04
47	SPAG1	cg04958845	2.14E-04
48	HLCS	cg16059256	2.14E-04
49	SNORD115-7	cg23841760	2.18E-04
50	UBE2J2	cg22670128	2.22E-04
51	ATL3	cg14589563	2.24E-04
52	NACC2	cg14350701	2.25E-04
53	CADM1	cg01850954	2.25E-04
54	COL11A2	cg20901684	2.25E-04
55	MLKL	cg27459981	2.32E-04
56	MSLN	cg06630425	2.39E-04
57	FOXP1	cg20088477	2.42E-04
58	ZFP41	cg06422464	2.46E-04
59	C14orf86	cg04836972	2.54E-04
60	FAM172A	cg25015043	2.58E-04
61	CNR1	cg19961480	2.60E-04

62	SSB	cg14157244	2.67E-04
63	MBD2	cg16200116	2.78E-04
64	RPH3AL	cg27489009	2.91E-04
65	KCTD15	cg20638467	2.93E-04
66	PRDM12	cg15700165	2.95E-04
67	UNC5B	cg18858343	3.05E-04
68	C17orf95	cg02318866	3.15E-04
69	AHNAK	cg20518446	3.16E-04
70	GPR142	cg20861314	3.19E-04
71	GTF3C1	cg00092389	3.21E-04
72	AHNAK2	cg13599499	3.32E-04
73	HEATR1	cg07700062	3.35E-04
74	CASP10	cg07754492	3.46E-04
75	GBP4	cg27285720	3.66E-04
76	RPH3AL	cg18771195	3.70E-04
77	INTS1	cg15976779	3.71E-04
78	NWD1	cg09500002	3.74E-04
79	SFXN3	cg07372974	3.78E-04
80	RGS19	cg14128040	3.98E-04
81	BACE2	cg14523602	3.99E-04
82	RPH3AL	cg04897931	4.07E-04
83	MTP18	cg22395002	4.07E-04
84	DPP10	cg26842423	4.14E-04
85	KIAA1274	cg04221624	4.17E-04
86	SCTR	cg22300806	4.24E-04
87	XKR4	cg12751432	4.43E-04
88	SNORD115-15	cg21751623	4.53E-04
89	MPRIP	cg12489964	4.53E-04
90	PRDM2	cg21584052	4.54E-04
91	MAD1L1	cg20446833	4.57E-04
92	TOP3B	ch.22.163059F	4.60E-04
93	PRPF39	cg12773498	4.69E-04
94	CSF1	cg14262716	4.73E-04
95	VSTM2A	cg02866628	4.89E-04
96	UFM1	cg04012509	4.91E-04
97	MCM6	cg10512742	5.00E-04
98	TBX1	cg16332936	5.03E-04
99	PPT2	cg12629909	5.04E-04
100	DDHD1	ch.14.624270R	5.05E-04
101	FBXL13	cg05423304	5.10E-04
102	PRKG1	cg25997796	5.14E-04
103	LOC150185	cg03400811	5.16E-04
104	HEBP1	cg03611265	5.28E-04
105	MIR662	cg26775123	5.35E-04

106	ACOX1	cg19198791	5.45E-04
107	VPS52	cg22562590	5.51E-04
108	XPNPEP3	cg12638490	5.56E-04
109	CSDAP1	cg11706835	5.74E-04
110	NRP1	cg25733480	5.74E-04
111	RPS27	cg00040380	5.76E-04
112	UNC80	cg24938830	5.77E-04
113	SSTR4	cg17586860	5.87E-04
114	NUP93	cg10586756	5.89E-04
115	CRCT1	cg01864564	5.92E-04
116	SLC16A9	cg06578117	5.94E-04
117	CPA2	cg15942185	6.04E-04
118	FAM43A	cg02072170	6.19E-04
119	EIF2AK4	cg03384128	6.23E-04
120	PLAGL1	cg07507918	6.29E-04
121	DGKD	cg02901644	6.42E-04
122	GOLSYN	cg08457298	6.43E-04
123	C5orf60	cg14851468	6.45E-04
124	MTMR7	cg12068916	6.59E-04
125	PDE4DIP	cg23977954	6.59E-04
126	PDXK	cg21870229	6.62E-04
127	SEMA3B	cg22093095	6.63E-04
128	SLC25A12	cg04337734	6.72E-04
129	B4GALT2	cg23001650	6.72E-04
130	KLHL24	cg22757384	6.82E-04
131	MLL4	cg15496871	6.99E-04
132	AGRN	cg07912402	7.03E-04
133	CEP120	cg01028844	7.03E-04
134	HSPA4	cg13778073	7.15E-04
135	FRZB	cg14693391	7.18E-04
136	WDR27	cg05066503	7.29E-04
137	DOT1L	cg21880720	7.38E-04
138	ELL3	cg16915619	7.39E-04
139	PRSS42	cg22550309	7.39E-04
140	EGFL8	cg21869704	7.51E-04
141	CTNNA2	cg20494682	7.55E-04
142	TSPYL5	cg09503853	7.55E-04
143	BCS1L	cg04970994	7.59E-04
144	MYH6	cg05241732	7.59E-04
145	BAZ2A	cg06607719	7.64E-04
146	SLC25A22	cg13749939	7.71E-04
147	YME1L1	cg01782320	7.77E-04
148	C22orf34	cg20744362	7.78E-04
149	FARS2	cg14029759	7.84E-04

150	BAI1	cg19721255	8.01E-04
151	SPIRE2	cg27072212	8.13E-04
152	SUSD3	cg05675356	8.29E-04
153	FAM155A	cg20343827	8.31E-04
154	PHRF1	cg03074210	8.32E-04
155	ARFGAP3	cg09861871	8.32E-04
156	SLC12A7	cg02350636	8.36E-04
157	WBSCR28	cg09117643	8.39E-04
158	TFB1M	cg27442478	8.42E-04
159	MYO9A	cg12195460	8.47E-04
160	ABCC5	cg10516336	8.49E-04
161	NMI	cg22664064	8.58E-04
162	HS6ST3	cg18748134	8.63E-04
163	AGAP1	cg07225241	8.66E-04
164	SAA2	cg17310354	8.68E-04
165	EEF1D	cg16873848	8.70E-04
166	IKZF5	cg22262889	8.72E-04
167	MTNR1B	cg06106126	8.72E-04
168	CLDN8	cg22540233	8.82E-04
169	SLIT3	cg07819331	8.97E-04
170	KIAA0408	cg17215061	9.00E-04
171	NLRP1	cg07121721	9.05E-04
172	KCNQ5	cg12967218	9.16E-04
173	CTNND2	cg19576843	9.17E-04
174	KLK10	cg03762081	9.18E-04
175	SLC9A9	cg14574489	9.24E-04
176	MAPK8IP3	cg02952494	9.28E-04
177	HCN3	cg02913804	9.28E-04
178	HAPLN2	cg23949901	9.32E-04
179	DARS	cg11309214	9.39E-04
180	TLE4	cg14241138	9.40E-04
181	MPPED2	cg23064082	9.50E-04
182	HCCA2	cg17139436	9.52E-04
183	DEFB121	cg19830128	9.54E-04
184	ADAMTSL2	cg00698646	9.56E-04
185	SRC	cg11210813	9.57E-04
186	PENK	cg16419235	9.63E-04
187	FAM155A	cg03333286	9.66E-04
188	RCSD1	cg16350225	9.78E-04
189	ACP1	cg00431236	9.85E-04
190	CDH23	cg22545027	9.89E-04
191	ARHGAP12	cg02775617	9.89E-04
192	CATSPERG	cg14675896	9.91E-04
193	SLC22A12	cg09797913	9.94E-04

Table S2: CpGs of *DPP10* and *SLC16A9* which are differentially methylated in the EtG+ group. Shown are the results obtained from EtG+ vs. controls by independent *t*-tests assuming unequal variances in R (version 3.2.2). The shown regions are obtained from USCS data base [1]. Bonferroni threshold for *DPP10* p_{bonf} = .0009 and for *SLC16A9* p_{bonf} = .002, respectively. FDR = false discovery rate.

Gene	CpG	Region	Methylation	*p*-Value	FDR
DPP10 (51 CpGs)	cg26842423	Body, Shore	decreased	.0004	.019
	cg00089091	Body	decreased	.011	.238
	cg23260456	Body	decreased	.022	.323
SLC16A9 (23 CpGs)	cg06578117	5'UTR, Enhancer=true	increased	.0006	.010
	cg02243437	1st Exon, 5'UTR, Enhancer=true	increased	.021	.175

1.2 Figures

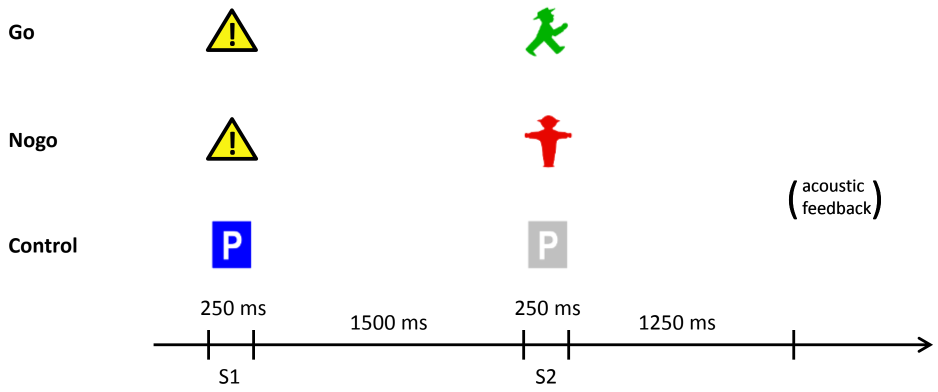

Figure S1: Illustration of the motivational Go/Nogo task (S1-S2 design).
All trial types (control, Go, Nogo) occurred with the same probability. Stimuli were presented for 250 ms. The interstimulus interval between S1 and S2 was 1500 ms, the intertrial interval was set to 3500 ± 500 ms. In two of the four task blocks, monetary incentives were used. Fast responses were rewarded by 10 ct. A fast response was defined individually using the 75th percentile of the reaction times in the previous task block. In turn, the same amount of money was removed if the participant did not react to a Go-stimulus within 1500 ms or when pressing the mouse button in control or Nogo trials. The participants received acoustic feedback for correct or incorrect responses (taken from Heinrich et al. [2], p.9.)

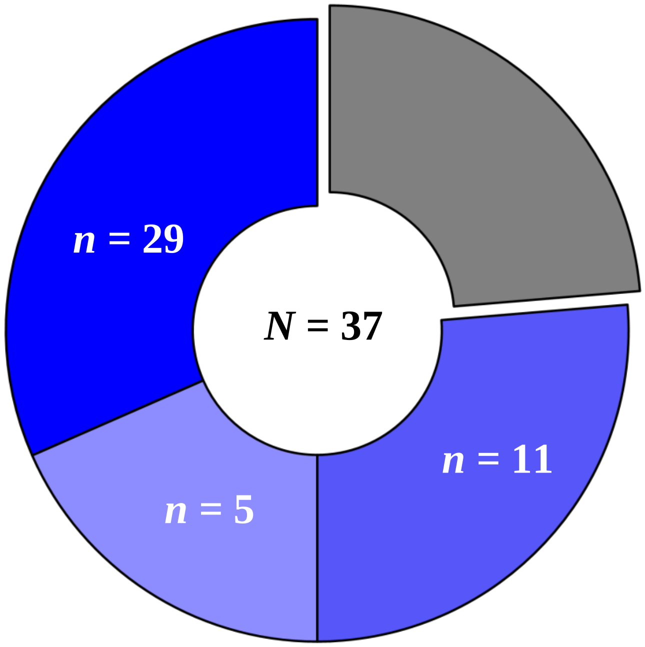

Figure S2: Classification of genes with relevance towards neuronal development according to GO-annotation [3, 4]. The whole circle represents 37 genes from Table S1 involved in nervous system development (19 %). Among these 37 genes are 29 genes, which are allocated to neurogenesis, as represented by entire blue part. The neurogenesis group is further divided into the subcategories neuronal differentiation (*n* = 11) and axon guidance (*n* = 5).

A

	Plate	Chip
PC1		
PC2		0.13
PC3	0.42	0.91
PC4		0.81
PC5		0.45
PC6	0.14	0.43
PC7		0.13
PC8		
PC9		0.23
PC10		0.61
PC11		
PC12		0.13
PC13		0.1
PC14		0.17
PC15		0.22
PC16		0.14
PC17		0.21
PC18		0.11
PC19		
PC20		0.1
PC21		
PC22		
PC23		
PC24		
PC25		
PC26		
PC27		
PC28		0.14
PC29		
PC30		

B

	Plate	Chip
PC1		
PC2		0.1
PC3		
PC4		0.1
PC5		0.16
PC6		0.2
PC7		0.15
PC8		0.14
PC9		0.14
PC10		0.13
PC11		0.11
PC12		0.16
PC13		
PC14		0.13
PC15		
PC16		
PC17		
PC18		
PC19		
PC20		
PC21		
PC22		0.13
PC23		
PC24		
PC25		
PC26		
PC27		0.1
PC28		
PC29		
PC30		

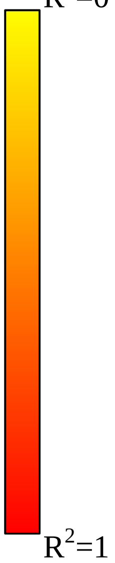

Figure S3. Principle component analysis results indicate technical biases due to plate and chip effects. Shown are R^2 values ≥ 0.1 before (**A**) and after (**B**) correction for 22 control probe PCs.

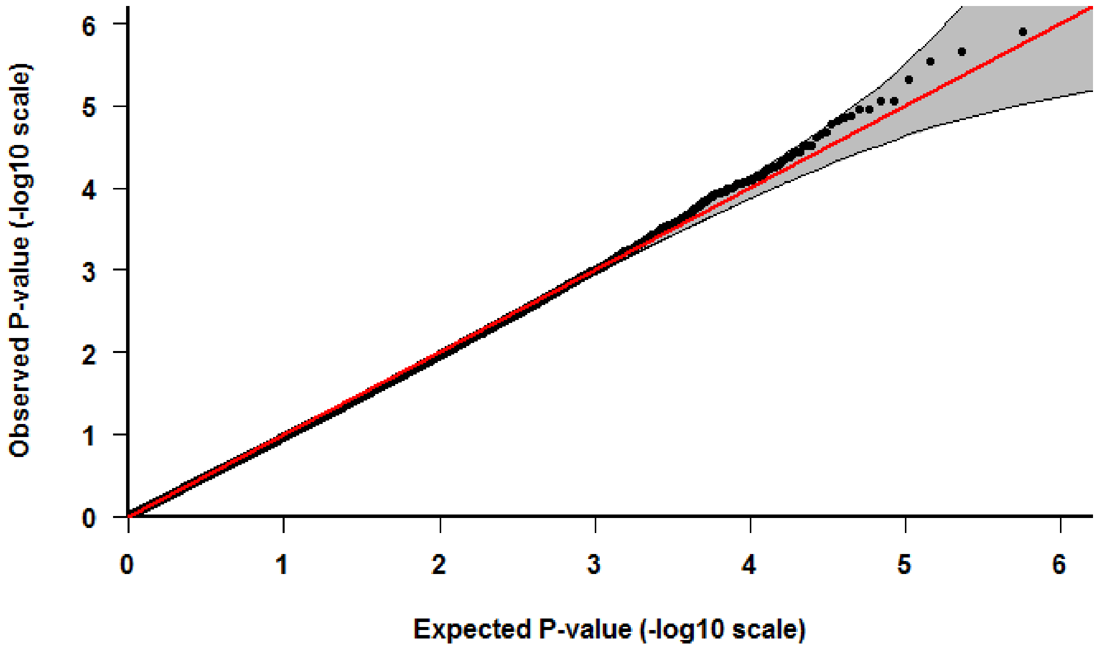

Figure S4: Quantile-quantile (QQ) plot of DNA methylation between EtG positive and control group. The x axis is the expected $-\log_{10}$ *p*-value, and the y axis is the observed $-\log_{10}$ *p*-value. λ= 0.93

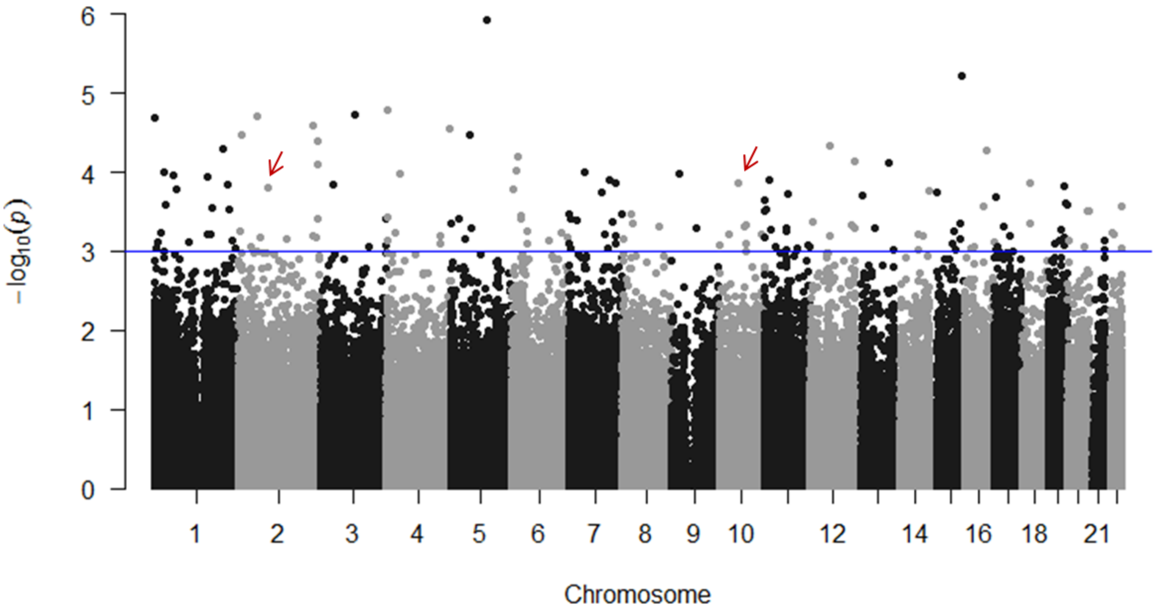

Figure S5: Manhattan plot showing differentially methylated positions plotted according to their chromosomal location with $-\log_{10}$ *p*-values. The blue line marks the threshold for genes considered in this study (Table S1). The red arrow on chromosome 2 indicates cg26842423 (*DPP10*) and on chromosome10 cg06578117 (*SLC16A9*), respectively.

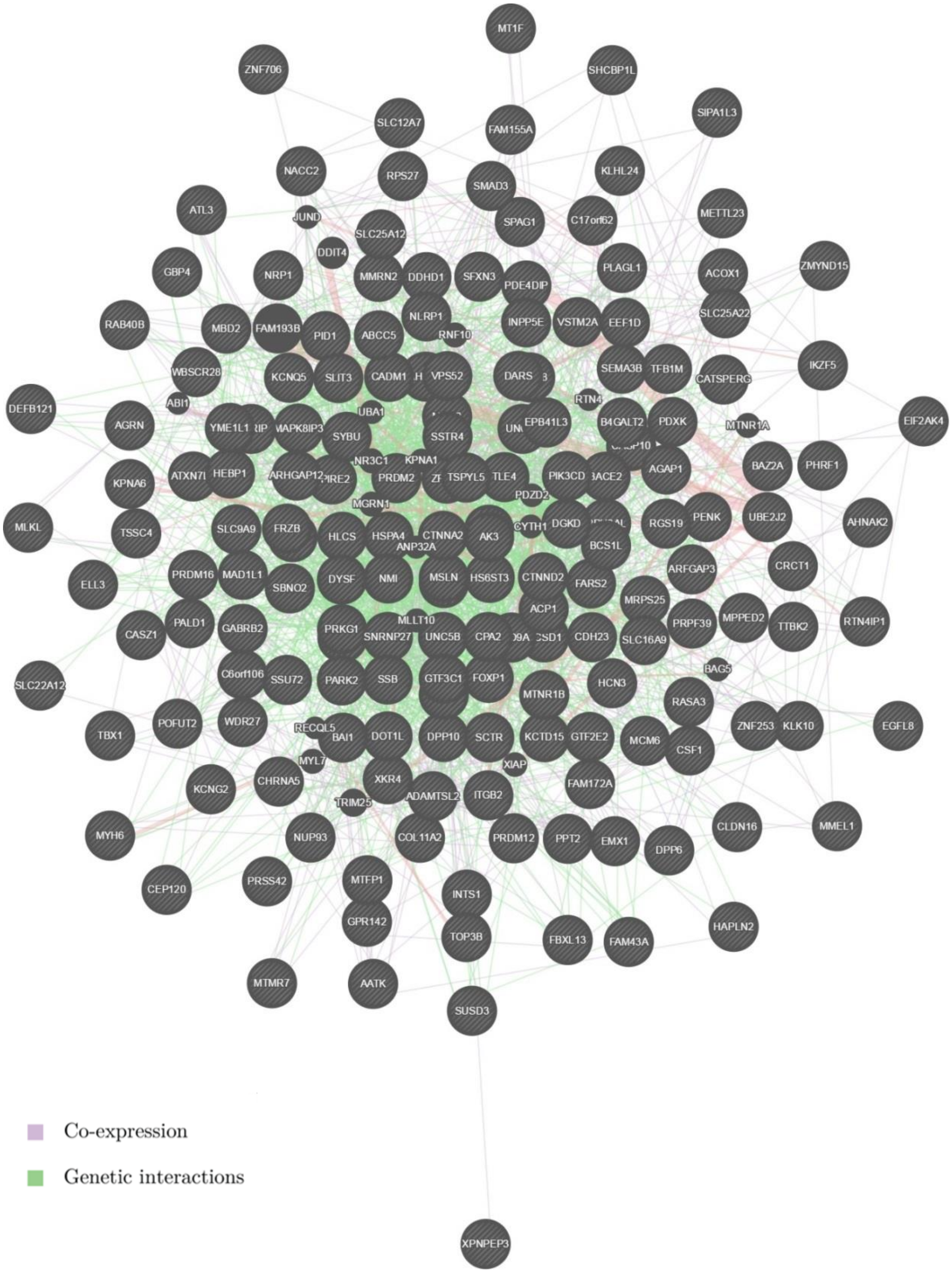

Figure S6: GeneMANIA network for all genes with at least one CpG differentially methylated in EtG+ vs. control children ($p < 10^{-3}$, Table S1).

References

1. Aken BL, Ayling S, Barrell D, Clarke L, Curwen V, Fairley S, *et al*. The Ensembl gene annotation system. *Database (Oxford)* 2016.
2. Heinrich H, Grunitz J, Stonawski V, Frey S, Wahl S, Albrecht B, *et al*. Attention, cognitive control and motivation in ADHD: Linking event-related brain potentials and DNA methylation patterns in boys at early school age. *Sci Rep* 2017; 7(1)**:** 3823.
3. Mi H, Huang X, Muruganujan A, Tang H, Mills C, Kang D, *et al*. PANTHER version 11: expanded annotation data from Gene Ontology and Reactome pathways, and data analysis tool enhancements. *Nucleic Acids Res* 2017; 45(D1)**:** D183-D189.
4. Thomas PD, Campbell MJ, Kejariwal A, Mi H, Karlak B, Daverman R, *et al*. PANTHER: a library of protein families and subfamilies indexed by function. *Genome Res* 2003; 13(9)**:** 2129-2141.
